# Uncovering predictors of myopia in youth: a secondary data analysis using a machine learning approach

**DOI:** 10.3389/fmed.2025.1595320

**Published:** 2025-10-21

**Authors:** Jiajia Liao, Zhijie Chen, Wanqing Jin

**Affiliations:** ^1^National Clinical Research Center for Ocular Diseases, Eye Hospital, Wenzhou Medical University, Wenzhou, China; ^2^Key Laboratory of Surface Modification of Polymer Materials, Wenzhou Polytechnic, Wenzhou, China

**Keywords:** myopia, machine learning, model, predictors, youth

## Abstract

**Introduction:**

Myopia is a multifactorial condition driven by an interplay of genetic predisposition and environmental triggers. This study aims to harmonize and analyze risk predictors from two distinct datasetsone historical and clinical, the other contemporary and behavioralto develop an integrated framework for myopia risk prediction.

**Methods:**

We analyzed two datasets: the Orinda Longitudinal Study of Myopia (OLSM), a 1995 US cohort (n≈500) with detailed ocular biometrics (e.g., spherical equivalent refraction, axial length) and lifestyle factors, and a 2022-2023 Chinese cross-sectional study (n=100,000) highlighting modern behaviors (e.g., screen time, posture). We employed multiple machine learning modelsincluding logistic regression, Explainable Boosting Machine (EBM), gradient boosting decision trees (GBDT) on OLSM, and deep neural networks (DNN) and XGBoost on the Chinese datasetto identify key predictors. Model interpretability was assessed using SHapley Additive exPlanations (SHAP). We also tested three ensemble strategies (sequential, averaging, transfer learning) to merge insights across the structurally divergent datasets.

**Results:**

Both datasets confirmed parental myopia as a universal risk factor and time spent outdoors as a protective factor. In the OLSM dataset, spherical equivalent refraction and parental myopia were the top predictors, with models achieving an AUC of up to 0.92. In the Chinese dataset, the DNN model achieved 71% accuracy, identifying screen time, posture, and parental history as major risk factors. Cross-dataset integration via transfer learning proved most effective, successfully amplifying features like outdoor activity and posture while retaining core behavioral predictors like screen time. This approach bridged the clinical depth of OLSM with the granular, modern lifestyle insights from the Chinese dataset.

**Discussion:**

Our analysis confirms the multifactorial nature of myopia, blending historical biological mechanisms with contemporary behavioral drivers. The study demonstrates a scalable strategy for global myopia risk prediction by adaptively integrating diverse datasets. While not yet a turnkey clinical tool, this work lays the groundwork for future multimodal risk-prediction frameworks that can bridge era-specific biases and harness machine learning to capture the evolving profile of myopia risk.

## 1 Introduction

Myopia, or nearsightedness, is emerging as one of the most prevalent refractive errors and eye problems worldwide. Between 1990 and 2023, the pooled prevalence among children and adolescents increased from approximately 24–36%, with projections reaching nearly 40% by 2050, affecting over 740 million young individuals worldwide (1). As the prevalence of myopia continues to rise, it poses a major public health challenge (1–4). In urbanized areas of East and Southeast Asia, which are considered developed regions, the prevalence of myopia is approximately 80–90% among young adults (5, 6). Moreover, many developed Western countries (mainly European countries) show substantially lower rates (20–40%) compared to East and South Asian countries, while less-developed regions and developing countries (with less intensive education systems) often have prevalence rates below 10% (2, 7, 8).

This issue is not only about the high prevalence but also the alarming rise in incidence. Recent epidemiological studies have demonstrated a dramatic increase in myopia incidence, mainly in urbanized populations of East Asia, where intense educational pressure and prolonged digital screen exposure have raised significant concerns (3, 4, 9). Myopia is not only a major cause of visual impairment but also a significant risk factor: approximately 49% of individuals with high myopia develop myopic macular degeneration, 3–8% experience retinal detachment, and the risk of glaucoma nearly doubles (10–12). Multiple studies have shown that myopia prevalence follows a distinct pattern, with the highest rates observed in urban female individuals (~20%), followed by urban male (~12%) and rural female individuals (~7%), and the lowest in rural male individuals (~5%). Multivariate analyses across these studies indicate that being a student or a professional significantly increases the risk of myopia, whereas rural residence is associated with a reduced risk. In addition, female individuals exhibit a modestly higher prevalence of myopia compared to male individuals (13–16). Therefore, understanding the risk factors and etiology of myopia is essential for developing effective prevention and intervention strategies.

The etiology and patho-mechanism of myopia are complex and multifactorial, involving an interplay between genetic predisposition and environmental exposures. Recent studies have highlighted two key biological theories explaining how myopia develops. The *compensatory mechanism theory* suggests that the eye grows in response to blurry images (defocus) to help improve vision, a process known as *emmetropization*. This has been shown in animal models using special lenses or visual deprivation to trigger eye growth (17, 18). The second major theory is the *dopamine hypothesis*, which explains that dopamine, a chemical released in the retina when exposed to bright light, helps slow down eye elongation. When dopamine levels are low, such as during prolonged time indoors or screen use, eye growth may continue unchecked, leading to myopia (19, 20). These core mechanisms are not only important for understanding the biology of myopia but also provide a strong foundation for using advanced statistics to understand each risk factor’s impact and predict the risk of myopia.

As previously mentioned, the development of myopia is influenced by various risk factors. Family history (parental myopia) has consistently been shown to be a strong predictor of myopia risk, with numerous studies stressing the heritability of axial elongation and refractive error (21, 22). Additionally, both modern and old environmental and behavioral factors—such as increased near work, reduced time outdoors, and higher screen time—have emerged as significant contributors to the development of myopia (23, 24). Recent studies with a focus on modern life have also emphasized the role of urbanization and socioeconomic standing, where variations in lifestyle behaviors correlate strongly with myopia prevalence (25, 26). These insights have significantly advanced the development of hybrid models that bring together clinical, genetic, and behavioral data to predict myopia risk with much greater accuracy.

Furthermore, recent improvements in imaging and biometric technologies have enabled the detailed quantification of ocular parameters, including anterior chamber depth (ACD), axial length (AL), and vitreous chamber depth (VCD). These precise and non-invasive measures provide objective biomarkers, and when combined with lifestyle and genetic data, these measures can clarify the mechanisms underlying myopia and its potential risk factors. Studies have demonstrated that ocular biometric parameters, in combination with genetic markers, explain a considerable portion of the variance in refractive outcomes (27, 28). This integrative approach has the potential to notify targeted interventions, particularly for high-risk pediatric populations, to mitigate the long-term burden of myopia-related complications.

In light of these developments, we bring together two available myopia studies conducted nearly 28 years apart: Zadnik et al.’s 1995–2000 U. S. longitudinal school-based study (≈500 children) (29), which offers high-precision ocular biometry (AL, spherical equivalent refraction (SPHEQ), ACD and VCD, lens thickness (LT)), detailed near-work and outdoor activity logs, and parental myopia status; and a 2022–2023 Chinese cross-sectional survey (≈100,000 young people) (30) emphasizing modern digital behaviors (daily TV/computer screen time, lying-down use, screen distance), homework and outdoor exercise frequency, residence type (urban or rural), socioeconomic factors, and parental myopia. Although these datasets differ in era, geography, design (longitudinal vs. cross-sectional), sample size, and variable types (continuous biometric measures vs. ordinal behavioral categories), we applied ensemble learning and transfer learning techniques to align and fuse their complementary strengths. While this integration is far from a turnkey clinical tool, it represents the first step toward harnessing heterogeneous, temporally separated studies and the power of machine learning to capture both biological and lifestyle drivers of myopia risk and to inspire future, more practical multimodal risk-prediction frameworks.

## 2 Methods

### 2.1 Data

In this study, we used two public databases to explore the main predictors of myopia in young populations. The first dataset (dataset-1) is based on Zadnik et al.’s (29) study on ocular predictors of juvenile myopia; the data are publicly available in the Kaggle database through https://www.kaggle.com/datasets/mscgeorges/myopia-study/data. The data were gathered from 554 children enrolled in the Orinda Longitudinal Study of Myopia (OLSM).

The second dataset (dataset-2) is based on Huang et al.’s (30) study on risk factors of myopia among young people. Their raw dataset is available through https://staticcontent.springer.com/esm/art%3A10.1038%2Fs41598-024-680765/MediaObjects/41598_2024_68076_MOESM2_ESM.xlsx (Table 1).

**Table 1:** Key features of the two datasets used in this study.

Aspect	Dataset-1 = Zadnik et al. (29). (OLSM)	Dataset-2 = Chinese Cross-Sectional Study (30)
Type of Study	Longitudinal cohort (5-year follow-up)	Cross-sectional
Study Period	1995 (baseline)	2022–2023
Location	USA	China
Sample Size	~500 children	~100,000 young people
Key Parameters	Clinical: Axial length (AL), spherical equivalent refraction (SPHEQ), anterior chamber depth (ACD), lens thickness (LT), vitreous chamber depth (VCD) Behavioral: Near-work hours (READHR, COMPHR, STUDYHR), outdoor activity (SPORTHR) Genetic: Parental myopia (MOMMY, DADMY)	Behavioral: Screen time (TV_Time_Daily, Computer_Time_Daily), homework time (ordinal), outdoor exercise frequency (ordinal) Environmental: Screen distance (TV/Computer), posture, residence type Genetic: Parental_Myopia (ordinal)
Unique Features	Longitudinal ocular biometrics (e.g., AL, SPHEQ) Composite near-work metric (DIOPTERHR)	Modern digital habits (e.g., lying-down screen use) Socioeconomic factors (e.g., father’s education)
Data Type	Numerical clinical/biometric variables Quantified hours/week for behaviors	Ordinal/categorical variables for behaviors Lacks ocular biometrics (e.g., axial length)

### 2.2 Approach to datasets

In a medical approach, data are gathered sequentially and the decisions made are dynamically adjusted according to recent information; the diagnosis, as well as any required interventions or medications, may change over time as the data evolve.

Our approach mirrored real-world clinical decision-making, where risk assessment is refined sequentially as additional patient data become available. Initially, we estimated a patient’s myopia risk using models trained on lifestyle-related features from Dataset-1 and Dataset-2, analogous to a clinician’s preliminary history-taking. Subsequently, Dataset-1 was augmented with paraclinical assessments (as ordered by an ophthalmologist), prompting a refinement of the initial risk prediction.

**Figure 1:**
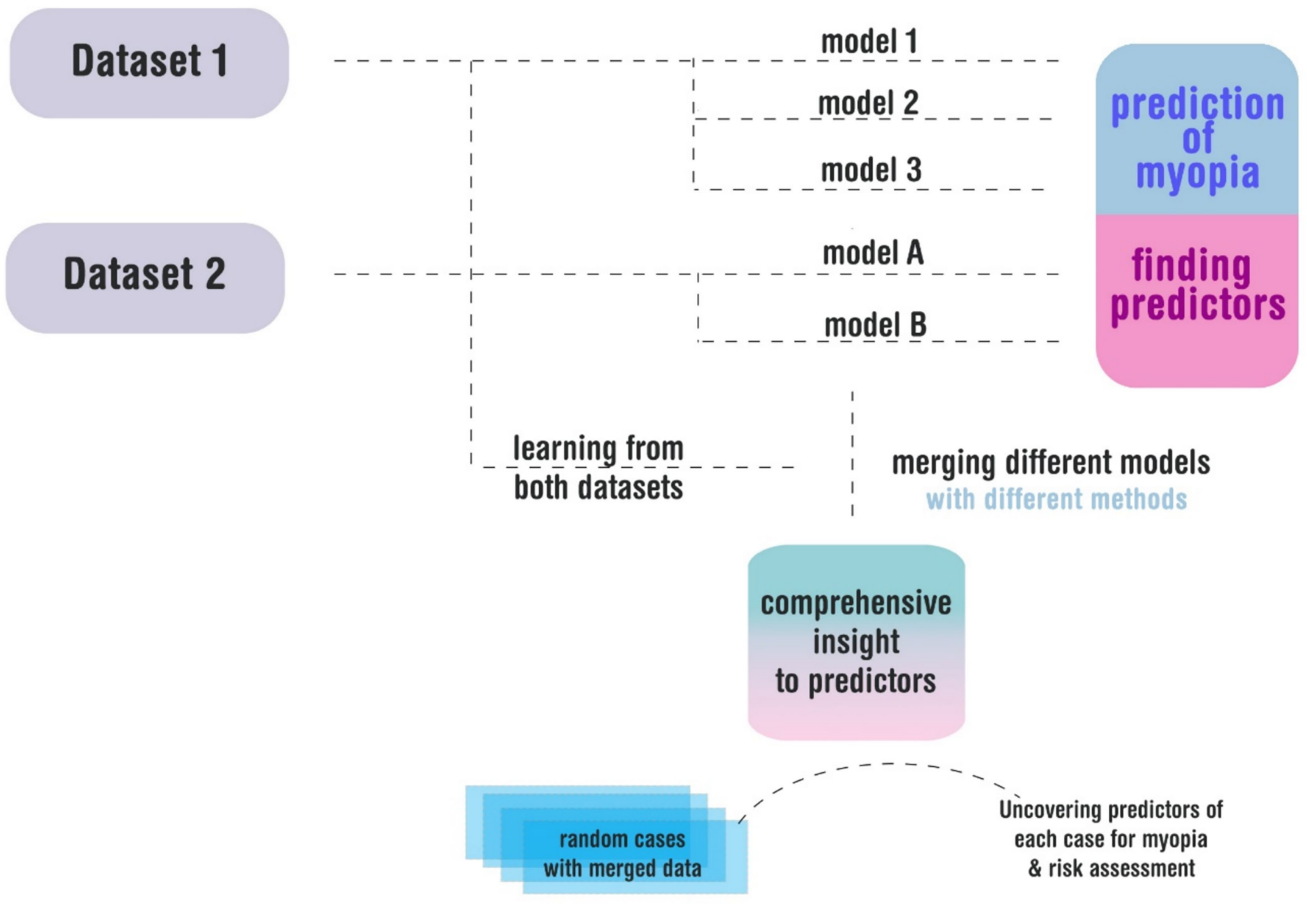
The graphical abstract illustrates our analytical pipeline, where distinct predictive models were trained separately on each dataset to identify myopia risk factors. These models were subsequently integrated into a unified framework capable of processing randomized case data, with input parameters standardized across both source datasets.

Finally, the two risk estimates were merged using distinct methodologies. Unlike conventional static approaches, our method was dynamic and holistic, closely resembling the iterative nature of clinical practice—where predictions are updated with new information (Figure 1).

### 2.3 Separate models training

The first model (model-1) employed on dataset-1 was a logistic regression classifier optimized using stochastic gradient descent (SGD). Logistic regression is a linear model suitable for binary classification tasks, while SGD iteratively updates the model’s parameters using small batches of data, making it efficient for large datasets. Before training, the input features are standardized using *StandardScaler* to ensure all features are on the same scale, which is crucial for the performance of gradient-based optimization methods. Hyperparameter tuning is performed using *GridSearchCV* with 5-fold cross-validation to identify the optimal regularization strength (alpha), which helps prevent overfitting and improves the model’s generalization capability. The model’s performance is evaluated using the Area Under the ROC Curve (AUC) score, a robust metric particularly useful for imbalanced datasets or when the focus is on the ranking quality of predictions.

For the second model (model-2) in the dataset-1 analysis, we employed an Explainable Boosting Machine (EBM), a glass-box Generalized Additive Model (GAM) with automated interaction detection. The model was optimized via grid search over five hyperparameters (max_bins, interactions, outer_bags, etc.). Categorical variables (*GENDER, PARENTMY*^1^) were explicitly encoded, and the model used cyclic gradient boosting with binning/smoothing to train additive functions for each feature and interaction. Performance was evaluated through 5-fold cross-validation using AUC scoring, with the hyperparameters tuned to balance interpretability (limited interaction terms) and predictive power (AUC-driven optimization). The final model retained all single features and top interaction pairs, weighted by their mean absolute contribution to the predictions.

For the third model (model-3) trained on dataset-1, we used a histogram-based gradient boosting decision tree (GBDT), implemented with the *HistGradientBoostingClassifier*. This model is particularly effective for handling large datasets and supports both numerical and categorical features. Hyperparameter tuning was performed using *GridSearchCV* with 5-fold cross-validation to optimize key parameters, including l2_regularization (for controlling overfitting), *max_bins* (for discretizing continuous features), and *min_samples_leaf* (for controlling the minimum number of samples required to split a leaf node). The model was configured with early stopping to prevent overfitting, a validation fraction of 0.15 to monitor performance, and a learning rate of 0.01 to ensure stable convergence. The categorical features were explicitly specified to ensure proper handling. The model’s performance was evaluated using the AUC score.

For the first model (model-a) on dataset-2, a deep neural network (DNN) was implemented to predict myopia using a structured input of categorical, ordinal, and numerical variables. The model consisted of four dense layers (128, 64, 32 neurons) with *ReLU* activation functions, batch normalization, and 30% dropout to prevent overfitting. The output layer utilized a sigmoid activation function for binary classification. The model was optimized using the Adam optimizer (learning rate = 0.001) and trained with binary cross-entropy loss for 50 epochs. Feature importance analysis was performed using SHapley Additive exPlanations (SHAP) to understand the impact of different predictors on myopia classification.

To consider another myopia prediction method, we utilized an enhanced XGBoost classifier as model-b on dataset-2, with 300 trees, a learning rate of 0.05, and a max depth of 10 to better capture complex patterns in the dataset. To reduce overfitting, we applied *min_child_weight* (3), subsample (0.8), and colsample_bytree (0.8). In addition, gamma (0.2) and L2 regularization (reg_lambda = 1.5) were incorporated for better generalization. The model was trained using a *log-loss* evaluation metric, and class imbalance was addressed with *scale_pos_weight* = 1. Feature importance was extracted after training to analyze the key predictors of myopia.

### 2.4 Model merging

#### 2.4.1 Approach A: sequential model merging

In this method, patient data were first managed separately for dataset-1 and dataset-2, ensuring that each model specialized in the specific data structure it was trained on. The first model trained on dataset-1 was used to generate a risk score, which was then passed to the second model trained on dataset-2 for final risk estimation. In this sequential merging approach, we retained the strengths of each dataset while capturing its domain-specific predictive insights. The implementation of this approach relied on Python libraries such as scikit-learn for model chaining, NumPy for numerical processing, and Pandas for data alignment. The primary rationale behind this approach was to retain dataset-specific nuances; while maintaining dataset-1’s clinical depth, dataset-2’s modern lifestyle focus was also preserved and resulted in improved predictive performance.

#### 2.4.2 Approach B: simple model output merging

This simple strategy involved running patient data through both models independently and then averaging their outputs with no superiority to obtain a final risk prediction. This method not only ensured a balance between the clinical insights from dataset-1 but also retained the large-scale behavioral trends captured by dataset-2. Similar to the previous method, merging was implemented using scikit-learn’s ensemble averaging techniques, with NumPy managing the arithmetic operations on model outputs. The rationale behind this approach was its simplicity and interpretability, as it allowed both models to contribute equally to the final prediction without requiring complex integration steps.

#### 2.4.3 Approach C: transfer learning

In this study, the model trained on dataset-1 served as a feature extractor, capturing core myopia-related representations. This pre-trained model was then fine-tuned on the reorganized dataset-2 to adapt to new patterns present in the modern dataset. The implementation was carried out using TensorFlow/Keras for deep learning-based feature transfer. This method was chosen due to its ability to enhance learned representations from dataset-1 while adapting to the behavioral and environmental shifts reflected in dataset-2, similar to previous approaches but more complex. Transfer learning enables better generalization, as the model benefits from both clinical depth knowledge and large-scale contemporary behavioral data simultaneously.

## 3 Results

### 3.1 Baseline characteristics of the two datasets

In this article, we primarily focus on the predictors used in this study, and the primary analysis is available in other articles (31). Baseline characteristics and primary analysis of the two datasets, which are provided in the Supplementary file in detail, and a brief review of the characteristics of each dataset is provided here. To understand the dataset-1 parameters, which are noted below, please refer to the Glossary of Terms and Abbreviations section, Supplementary Table 1; Supplementary Figure 1.

The baseline characteristics of dataset-1 (OLSM) provided important insights into the study population. The distribution of the MYOPIC parameters indicated a higher proportion of participants without myopia, aligning with previous observations. Ocular biometric variables such as ACD, SPHEQ, AL, and VCD exhibited normal or slightly skewed distributions, reflecting expected variations in eye structure (Supplementary Figure 2). In addition, the participant’s age at study entry (AGE) demonstrated a normal distribution.

Time-related variables, including time spent reading for pleasure (READHR), time spent reading/studying for school assignments (STUDYHR), time spent engaging in sports/outdoor activities (SPORTHR), and time spent watching television (TVHR), exhibited right-skewed distributions, indicating that the majority of the participants engage in moderate levels of these activities, with a smaller subset displaying higher durations (Supplementary Figures 2, 3). The myopia rate trends across these variables suggested potential associations with reading and screen time, emphasizing the importance of lifestyle factors in myopia development.

Categorical variables such as gender, parental myopia [especially if the patient’s mother has a history of myopia (MOMMY), if the patient’s father has a history of myopia (DADMY), or whether one of the patient’s parents or both have a history of myopia (PARENTMY)], and their respective myopia rates provide additional context. Notably, the myopia rate was higher among participants with both myopic parents, reinforcing the hereditary influence of myopia (Supplementary Figure 3).

These findings highlight the diversity in ocular characteristics and lifestyle habits within the cohort, emphasizing the significance of considering these factors in myopia-related studies. This baseline analysis provides a robust foundation for further investigations into risk factors and outcomes.

In the context of the baseline characteristics of dataset-2, the distribution of categorical variables revealed significant patterns that aligned with myopia prevalence trends (Supplementary Figure 4). Gender distribution was relatively balanced, while age showed a higher frequency in younger groups but a progressive increase in myopia with age. Residence type distribution indicated a higher proportion of individuals from urban areas, who also exhibited a higher myopia rate (Supplementary Figure 4). Family income distribution was skewed toward lower-income groups, although higher-income individuals showed slightly increased myopia prevalence. Parental education levels varied, with higher education levels associated with greater myopia rates. Behavioral factors, such as lying down or moving while using the eyes, excessive screen time, and close viewing distances, showed a declining frequency but a rising myopia trend, indicating their role as risk factors. In contrast, outdoor exercise, proper posture when reading or using a screen, and adequate sleep showed an inverse relationship with myopia but were less frequent in the dataset (Supplementary Figure 4). In conclusion, the data distribution highlights that while some risk factors are common, their correlation with myopia suggests a need for lifestyle interventions to mitigate its prevalence.

### 3.2 Primary model training on the datasets

The models associated with dataset-1 are labeled as Model-1, Model-2, and so on. During the training of these models, certain parameters were combined to simplify the model inputs. DIOPTERHR is a composite measure of near-work activity burden, calculated as 3 × (READHR + STUDYHR) + 2 × COMPHR + TVHR, where READHR and STUDYHR represent hours spent reading and studying (the highest accommodative demand), COMPHR is computer use (moderate demand), and TVHR is television viewing (the lowest demand). The weights reflect the typical working distances of each activity, aligning with physiological models of accommodative effort based on diopter demand (please visit Supplementary Table 1).

Similarly, the parameter PARENTMY was derived as the sum of DADMY and MOMMY. These consolidations were implemented to streamline the input structure and enhance computational efficiency.

**Figure 2:**
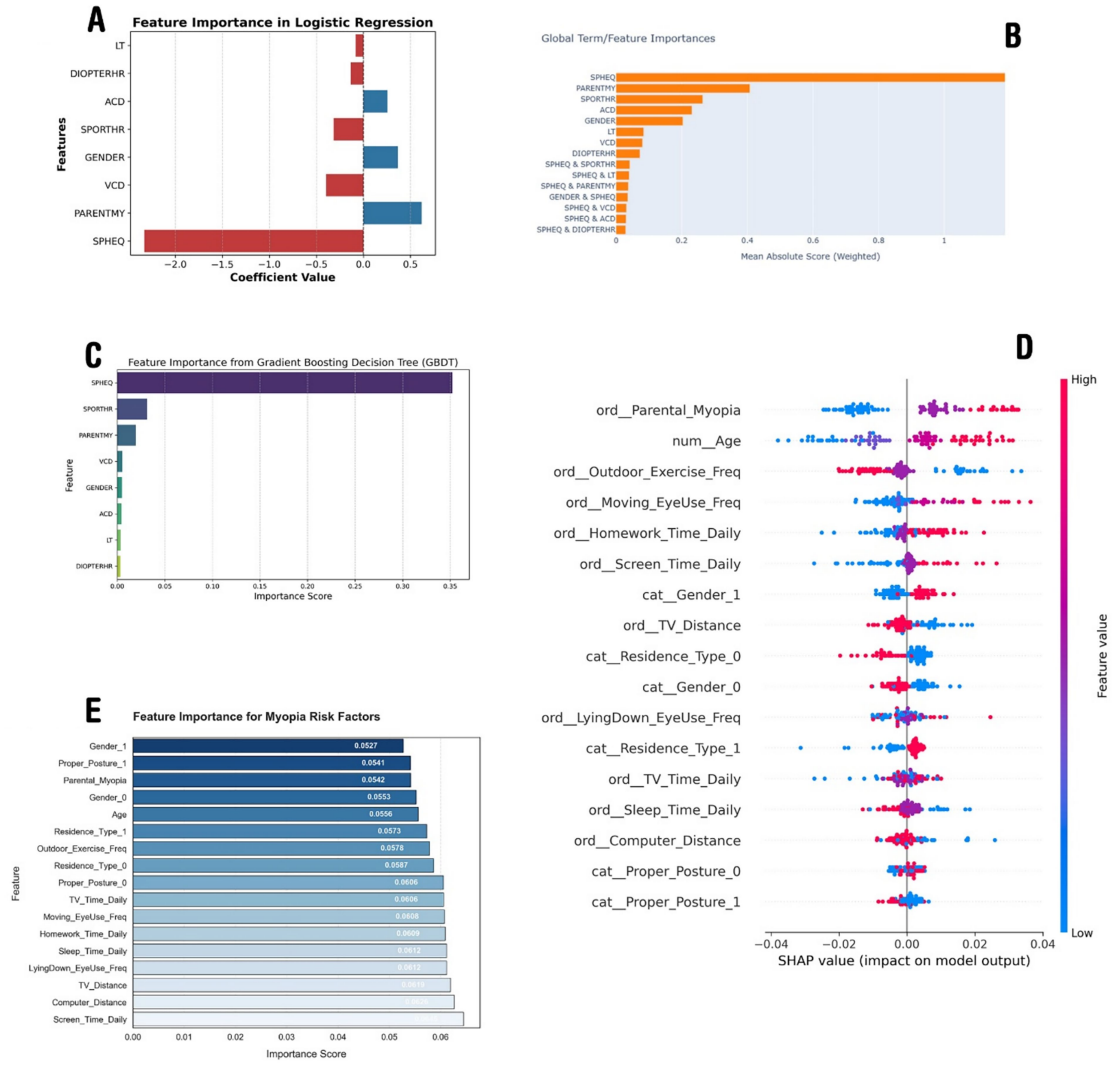
The importance analysis results of the models. **(A)** Demonstrates feature importance according to the linear regression model trained on dataset-1. **(B)** Demonstrates the important parameters according to the EBM trained on dataset-1. **(C)** Illustrates features importance according to the GBDT trained on dataset-1. **(D)** Depicts most important features according to the SHAP analysis of the DNN model trained on dataset-2, and **(E)** demonstrates the XGBoost model trained on dataset-2.

For Model-1, trained on dataset-1, a logistic regression model with SGD optimization was used and fine-tuned using grid search, leading to the selection of the optimal regularization parameter (alpha = 0.001). Model performance was evaluated using the AUC score, ensuring a robust assessment of class distinction capability. The feature importance analysis (as shown in Figure 2A) showed SPHEQ as the most influential negative predictor, followed by VCD and SPORTHR, while PARENTMY and GENDER emerged as the strongest positive predictors. Features with positive coefficients (blue) contributed positively to the predicted outcome, while those with negative coefficients (red) had an inverse effect. The model achieved a training AUC of 0.890 and training accuracy of 0.896, while cross-validation results showed an AUC of 0.875 and accuracy of 0.892, demonstrating strong generalization performance.

These model outputs align with myopia research, confirming SPHEQ as the strongest negative predictor, reflecting the severity of myopia. Parental history (PARENTMY) also shows a strong correlation, highlighting genetic influence. Outdoor activity (SPORTHR) is negatively associated, supporting its protective effect. Conversely, VCD is a key positive predictor, linking axial elongation to myopia progression. Gender (GENDER) also shows a positive effect, possibly due to a higher prevalence in female individuals. Notably, DIOPTERHR is not a dominant predictor, suggesting that genetic and axial growth factors play a more significant role (Figure 2A).

The second model (model-2) trained on dataset-1 was the Explainable Boosting Machine (EBM), which identified SPHEQ (spherical equivalent refraction) as the strongest predictor of myopia, contributing nearly twice the importance of the second-ranked feature, PARENTMY (parental myopia history). Behavioral factors such as SPORTHR (sports hours) and biometric measures (ACD, VCD) showed moderate predictive power, while interaction terms (e.g., SPHEQ & SPORTHR, SPHEQ & PARENTMY) revealed synergistic effects, collectively explaining 40% of the model’s predictive capacity (Figure 2B). The model achieved strong discrimination (AUC: 0.92 ± 0.03), with SPHEQ-driven interactions highlighting how refractive error modifies the impact of environmental factors, such as sports activity, on myopia risk.

The third model (model-3), a histogram-based GBDT, after the hyperparameter tuning process and finding the optimal configuration for the GBDT model, was then used to evaluate feature importance. The feature importance analysis, visualized in Figure 2C, revealed that SPHEQ and SPORTHR were the most influential features, with importance scores of approximately 0.35 and 0.25, respectively. Other features, such as PARENTMY, VCD, and GENDER, showed moderate importance, while ACD, LT, and DIOPTERHR had relatively lower impact on the model’s predictions. These results provide valuable insights into the key drivers of the model’s decision-making process and highlight the most significant features for further analysis or model refinement.

In the context of dataset-2, the first model (model-a) employed was a deep neural network. After tuning and training the model, the model reached a validation accuracy of 0.87. Using the parameters of the model and the SHAP library, the effect of each parameter on the model output was calculated.

A DNN was implemented on dataset-2 as model-a, which achieved an accuracy of 71.32%, showing a slight improvement over the XGBoost model (model-b discussed later). The SHAP analysis revealed that the most influential factors were parental myopia, age, outdoor exercise frequency, moving eye-use frequency, and homework time, emphasizing the role of both genetic and environmental factors in myopia development. Higher feature values for screen time, computer distance, and TV distance were also found to significantly impact the predictions. The SHAP summary plot (Figure 2D) further illustrated the relationship between feature values and their influence on the model’s decision-making, confirming the importance of lifestyle habits in myopia prediction.

The second model (model-b), trained on dataset-2, was the improved XGBoost model that achieved a test accuracy of 66.88%, with a precision of 71% for myopic cases (class 1) and 30% for non-myopic cases (class 0). The recall for myopia detection was high (89%), indicating that the model effectively identified myopic individuals but struggled with false positives. The most influential features included screen time, computer distance, TV distance, lying down while using the eyes, and sleep duration, highlighting the impact of lifestyle habits on myopia (Figure 2E). Despite improvements, the model’s overall balance between precision and recall suggests that further tuning or alternative approaches might be needed for better classification.

### 3.3 Understanding model-related risk factors

The two datasets share core similarities regarding demographic variables (age, gender), parental myopia history, and behavioral factors (outdoor activity, near-work hours), enabling partial harmonization of risk predictors such as genetic predisposition and environmental exposure. Both capture critical myopia drivers but differ structurally: the dataset-1 cohort (USA, 1995, *n* = 500) provides longitudinal data and detailed ocular biometrics (axial length, spherical equivalence), while the Chinese cross-sectional dataset (2022–2023, *n* = 100,000) emphasizes modern lifestyle factors (screen time, posture) with ordinal coding. Key challenges include reconciling numerical (dataset-1) and ordinal (dataset-2) variables, temporal/geographic biases (pre-digital vs. tech-era behaviors), and study design mismatch (cohort vs. cross-sectional). However, synergies exist in leveraging the Chinese dataset’s scale to identify broad risk patterns and dataset-1’s clinical depth to model biological mechanisms. Techniques such as transfer learning could merge their strengths, validating universal predictors (e.g., parental myopia, outdoor activity) while accounting for era-specific confounders to build a global myopia risk framework integrating genetic, behavioral, and clinical dimensions.

The analysis of myopia risk factors across the two datasets, dataset-1 and dataset-2, revealed both shared and distinct predictors influenced by the dataset structure and temporal context. Figures 2A–C (dataset-1) highlight the importance of traditional ocular biometric factors (e.g., spherical equivalence, axial length) and parental myopia, emphasizing biological determinants. Conversely, Figures 2D–E (dataset-2) prioritize modern lifestyle behaviors (e.g., screen time, posture, near-work distance) as dominant predictors, reflecting the digital-era impact on visual health. While both datasets confirm the significance of parental myopia and outdoor activity, the Chinese dataset’s ordinal feature encoding provides a more granular behavioral assessment, allowing refined risk modeling. The SHAP analysis (Figure 2D) further illustrated nuanced feature interactions in contemporary lifestyles, contrasting with the GBDT feature importance (Figure 2C) that underscored traditional refractive parameters. This comparison suggests that integrating both datasets using transfer learning or hybrid modeling could enhance myopia risk prediction by combining clinical depth (dataset-1) with large-scale behavioral insights (dataset-2), ultimately improving prevention strategies across different populations and eras.

### 3.4 Merging models and their risk assessments

**Figure 3:**
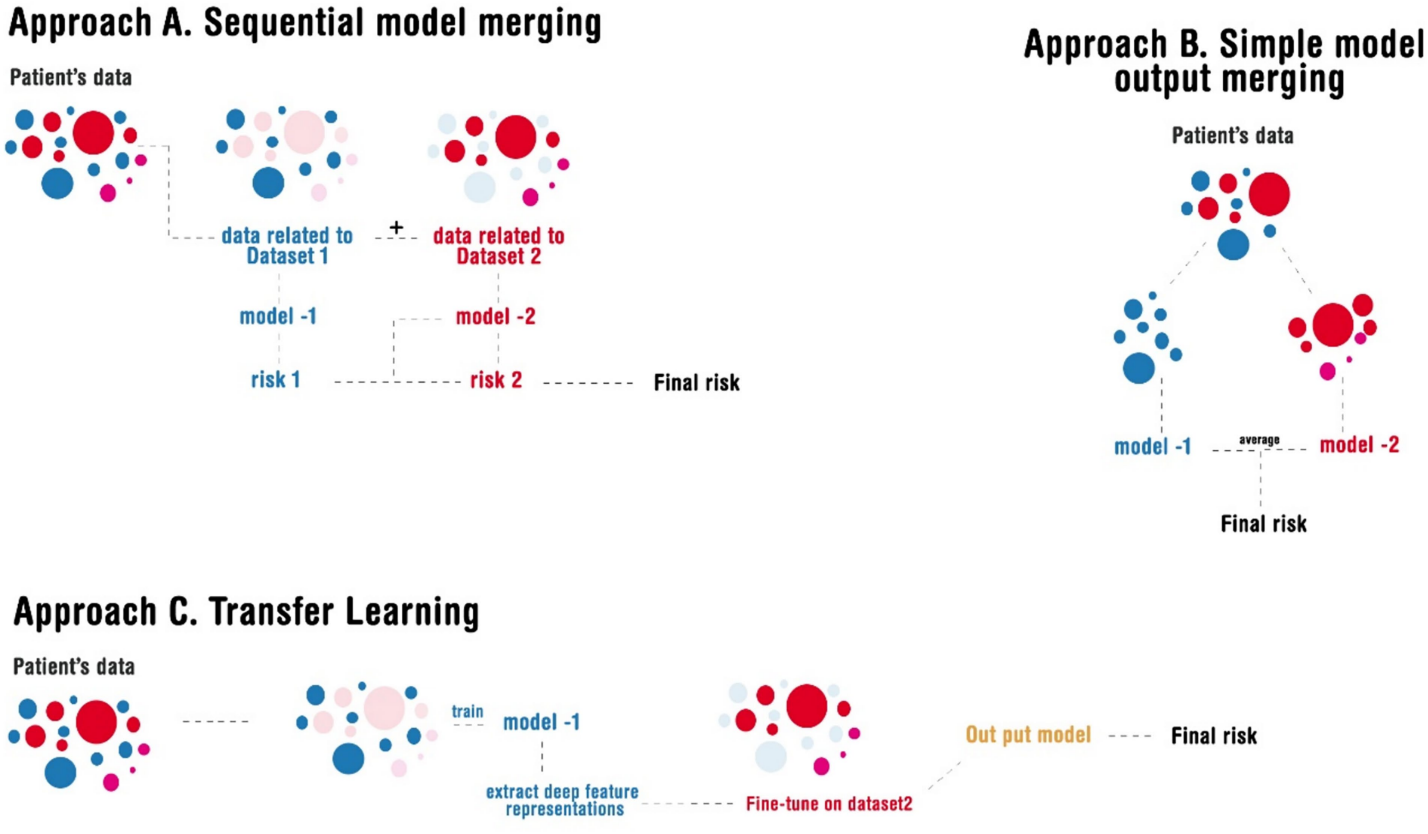
Illustration of the different approaches implemented in merging the models.

**Figure 4:**
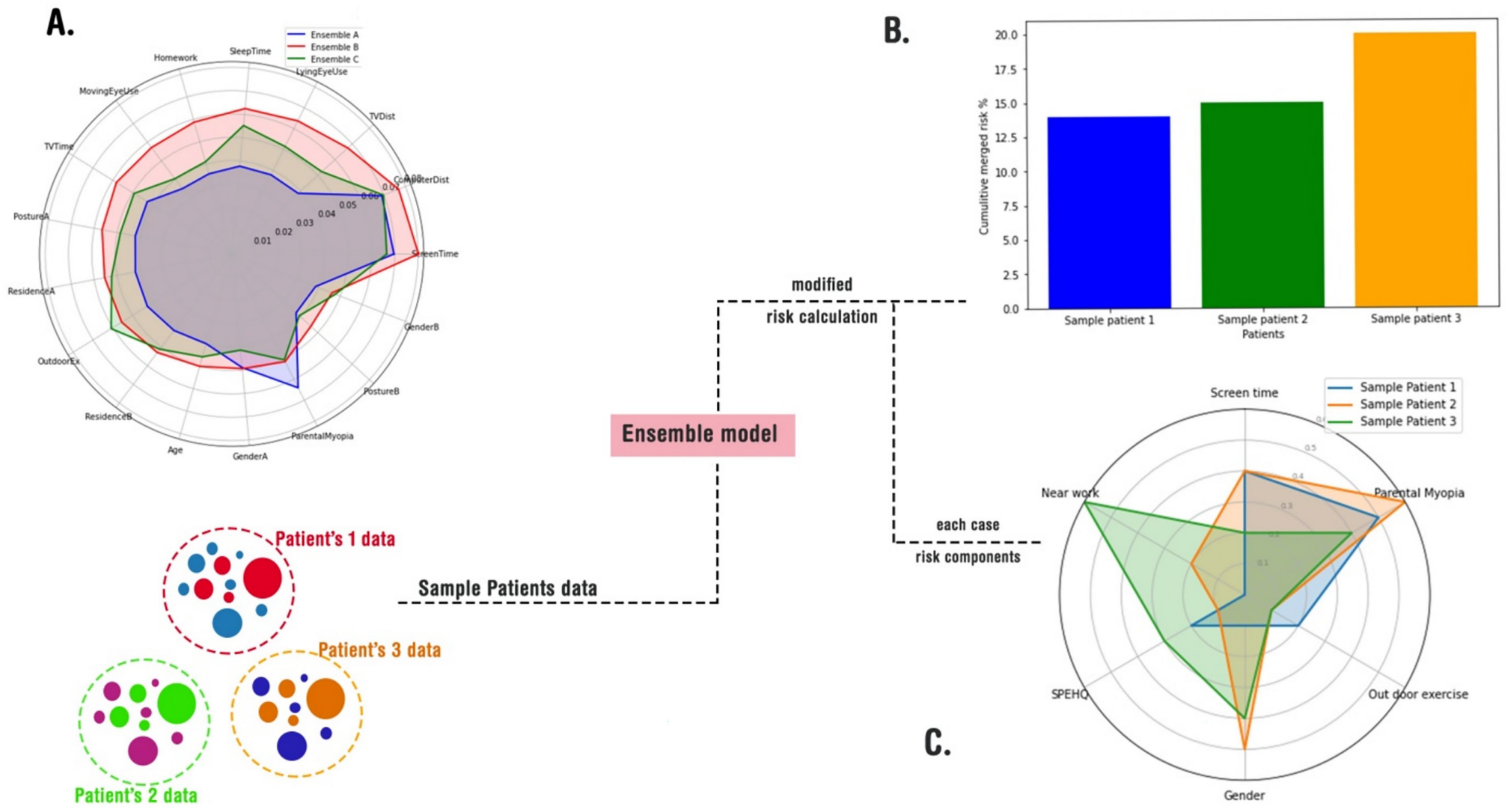
A schematic usage of the merge models in a real-world-like scenario. **(A)** A radar plot of cumulative risks according to cases; **(B)** feeding the merged models with random cases and calculating their total risks; **(C)** depicting an explainable risk assessment for each case.

As discussed in the method, we implemented three approaches for merging these models (Figure 3). The radar plot highlights variations in feature importance across the three ensemble approaches, revealing how different model merging strategies influence the final output. Sequential merging (Ensemble A - blue) preserved the dominance of *ScreenTime* and *Near work,* reflecting dataset-specific strengths but slightly underrepresenting secondary features such as *OutdoorEx* and *Residence B* (in an urban area). The simple averaging approach (Ensemble B - red) balanced contributions from both models, ensuring *LyingEyeUse* and *TVDist* (Near Work) gained more prominence, although it lacked the adaptability to refine feature relationships deeply. Transfer learning (Ensemble C - green) exhibited the most flexibility, redistributing feature weights significantly by amplifying *OutdoorEx* and *Parental myopia* while maintaining core influences such as *ScreenTime* and *ComputerDist* (Near works), suggesting that it adapts better to evolving patterns. Overall, sequential merging maintains dataset-driven strengths, simple averaging ensures fair representation, and transfer learning offers the highest adaptability, making it a robust choice for integrating diverse datasets with dynamic trends (Figure 4A). A sample of how the ensemble model works is shown in Figures 4B,C.

While merging models is a valuable approach for integrating diverse risk factors and calculating a comprehensive risk score for each patient, it is not without limitations. One significant concern is that the output of such a combined model has not been validated in real-world scenarios. The final risk score is essentially a composite of outputs from multiple models, each with distinct methodologies and assumptions. This raises questions about the robustness and generalizability of the results. Furthermore, the absence of validation against a control or test group underscores the need for further investigation to assess the reliability and accuracy of this method.

## 4 Discussion

Our integrated model framework highlights that both genetic and environmental factors play pivotal roles in myopia development. Conѕiѕtent with previouѕ ѕtudies (32–34), the merged modelѕ strongly emphaѕize parental myopia and ocular biometric indiceѕ aѕ key determinantѕ, highlighting the ѕtrong role of hereditary and anatomical influenceѕ. At the ѕame time, lifestyle and environmental factorѕ—such as ѕcreen time, near-work activitieѕ, and leѕѕ time ѕpent outdoorѕ—ѕtand out aѕ ѕignificant riѕk factorѕ, aligning with recent findingѕ from large-ѕcale croѕѕ-ѕectional ѕtudieѕ in urbanized ѕettingѕ. (35–37). This duality in risk profiles reinforces the necessity of multifaceted prevention strategies that address both intrinsic and extrinsic determinants of myopia.

When we compare our findings with earlier studies, it is interesting to see that our sequential merging and transfer learning methods produce feature importance profiles that accurately reflect the mix of clinical and behavioral differences seen across various populations. For example, while older models mostly focus on biometric predictors such as spherical equivalent refraction and axial length (28, 38, 39), the inclusion of modern lifestyle factorѕ—such as digital device uѕe and time ѕpent outdoorѕ—through enѕemble averaging and transfer learning haѕ really ѕhifted the ѕpotlight toward behaviorѕ we can actually change (33, 40). Such a conjunction of clinical and behavioral insights from different datasets and studies provides a more holistic view of myopia risk, consistent with the growing body of literature advocating for integrated risk assessment models.

Moreover, the overlapping radar plot of enѕemble feature importance illuѕtrateѕ that different model merging ѕtrategieѕ can yield complementary inѕightѕ. The XGBooѕt-dominant approach preѕerveѕ ѕtrong ѕignalѕ from eѕtabliѕhed clinical predictorѕ, while the DNN-dominant method accentuateѕ modern lifestyle influenceѕ. The approach emphasizing traditional models via the GBDT and logistic regression appears to balance these aspects effectively, suggesting that model integration can be tailored to optimize predictive performance depending on the population and context (41, 42). Our results align well with recent meta-analyses that recommend a hybrid model for global myopia risk prediction, especially in reconciling discrepancies between historical and contemporary data sources (43).

In conclusion, the synthesis of multiple modeling approaches underscores the multifactorial nature of myopia, where genetic, biometric, and environmental factors converge to determine disease risk. Our findings advocate for the adoption of integrated predictive models that combine the strengths of different methodologies to yield a comprehensive risk assessment tool. Such models not only enhance our understanding of the complex interplay between various risk factors but also pave the way for personalized interventions aimed at curbing the myopia epidemic. Future research should focus on validating these hybrid approaches across diverse populations and exploring their potential for real-time risk stratification and clinical decision support.

### 4.1 Limitations of this study

This study has several limitations and challenges, including differences in populations, time periods, and predictors. Firstly, as dataset-1 and dataset-2 are derived from different populations, it is essential to keep in mind that risk factors and baseline risk levels may vary between them. On top of that, temporal differences—such as shifts in diagnostic criteria or environmental factors over time—could also play a role in shaping the outcomes. Another thing to note is that while some predictors were common across the datasets, others were unique to specific datasets. This meant we had to carefully think through how to harmonize them or use a sequential modeling approach to handle them properly. Even with these limitations and challenges, we made a conscious effort to actively address and thoughtfully consider each issue throughout the study to minimize its potential impact on the results.

## 5 Conclusion

Our study highlights the complex, multifactorial nature of myopia, combining genetic, biometric, and lifestyle predictors using advanced modeling techniques. By bridging historical clinical insights with modern behavioral trends, we showcase the effectiveness of ensemble and transfer learning methods in improving risk assessment. This holistic approach provides a scalable framework for analyzing two distinct datasets with different parameters. Although our method prioritized merging the datasets and understanding the shared risk among their data, it will be crucial to validate these models across diverse populations to strengthen real-time risk stratification and support better clinical decision-making.

## Data Availability

The original contributions presented in the study are included in the article/Supplementary material, further inquiries can be directed to the corresponding author. Dataset-1: refers to data retrieved from Zadnik et al. study (29) which is also called OLSM, dataset-2: refers to data retrieved from Huang et al. study (30).

## Footnotes

^1^For detailed definitions of the variables, refer to the Glossary of Terms and Abbreviations section and Supplementary Table 1.

## Glossary

OLSM: Orinda Longitudinal Study of Myopia
SPHEQ: Spherical Equivalent Refraction
VCD: Vitreous Chamber Depth
SPORTHR: sports/outdoor activities
READHR: time spent reading
EBM: Explainable Boosting Machine
GBDT: Gradient Boosted Decision Trees
ACD: including anterior chamber depth
AL: axial length
VCD: vitreous chamber depth
LT: lens thickness
SGD: stochastic gradient descent
AUC: Area Under the ROC Curve
GAM: glass-box Generalized Additive Model
STUDYYEAR: Year the patient entered the study (Numerical, year)
MYOPIC: Myopia within the first five years of follow-up (Categorical, 0 = No; 1 = Yes)
AGE: Age at first visit (Numerical, years)
GENDER: Gender (Categorical, 0 = Male; 1 = Female)
SPHEQ: Spherical Equivalent Refraction (Numerical, diopter)
AL: Axial Length (Numerical, mm)
ACD: Anterior Chamber Depth (Numerical, mm)
LT: Lens Thickness (Numerical, mm)
VCD: Vitreous Chamber Depth (Numerical, mm)
SPORTHR: Time spent engaging in sports/outdoor activities (Numerical, hours per week)
READHR: Time spent reading for pleasure (Numerical, hours per week)
COMPHR: Time spent playing video games/working on the PC (Numerical, hours per week)
STUDYHR: Time spent reading/studying for school assignments (Numerical, hours per week)
TVHR: Time spent watching television (Numerical, hours per week)
DIOPTERHR: Composite of near-work activities (Numerical, hours per week)
MOMMY: Myopic Mother in patients familial history (Categorical, 0 = No; 1 = Yes)
DADMY: Myopic Father in patients familial history (Categorical, 0 = No; 1 = Yes)
PARENTMY: Sum of parents history of myopia (MOMMY + DADMY, Numerical)
