# Supplementary material for: Uncovering predictors of myopia in youth: a secondary data analysis using a machine learning approach
Source: Front Med (Lausanne). 2025 Oct 21;12:1595320. doi: 10.3389/fmed.2025.1595320 (PMC12583174; doi:10.3389/fmed.2025.1595320)
Supplement: Supplementary file 1 [file Data_Sheet_1.docx]

| **No.** | **Description** | **Value/Unit** | **Variable Name** |
| --- | --- | --- | --- |
| 1 | Year the patient entered the study | Numerical (year) | STUDYYEAR |
| 2 | Myopia within the first five years of follow-up | Categorical (0 = No; 1 = Yes) | MYOPIC |
| 3 | Age at first visit | Numerical (years) | AGE |
| 4 | Gender | Categorical (0 = Male; 1 = Female) | GENDER |
| 5 | Spherical Equivalent Refraction | Numerical (diopter) | SPHEQ |
| 6 | Axial Length | Numerical (mm) | AL |
| 7 | Anterior Chamber Depth | Numerical (mm) | ACD |
| 8 | Lens Thickness | Numerical (mm) | LT |
| 9 | Vitreous Chamber Depth | Numerical (mm) | VCD |
| 10 | Time spent engaging in sports/outdoor activities | Numerical (hours per week) | SPORTHR |
| 11 | Time spent reading for pleasure | Numerical (hours per week) | READHR |
| 12 | Time spent playing video games/working on the PC | Numerical (hours per week) | COMPHR |
| 13 | Time spent reading/studying for school assignments | Numerical (hours per week) | STUDYHR |
| 14 | Time spent watching television | Numerical (hours per week) | TVHR |
| 15 | Composite of near-work activities | Numerical (hours per week) | DIOPTERHR |
| 16 | Myopic Mother in patients familial history | Categorical (0 = No; 1 = Yes) | MOMMY |
| 17 | Myopic Father in patients familial history | Categorical (0 = No; 1 = Yes) | DADMY |
| 18 | Sum of parents history of myopia | MOMMY + DADMY (Numerical) | PARENTMY |

SupTable1 - Parameters of dataset -1 (OLSM), Here is to note; DI𝑂𝑃𝑇𝐸𝑅𝐻𝑅 = 3∗(𝑅𝐸𝐴𝐷𝐻𝑅+𝑆𝑇𝑈𝐷𝑌𝐻𝑅)+2∗𝐶𝑂𝑀𝑃𝐻𝑅+𝑇𝑉𝐻𝑅^[[1]](#footnote-1)^


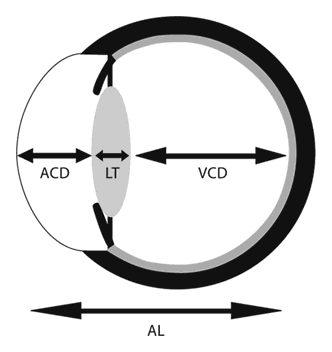


Sup fig1. Chamber Depth (ACD), Lens Thickness (LT), Vitreous Chamber Depth (VCD), and Axial Length (AL). These parameters are critical for assessing eye anatomy and are often used in studies related to refractive errors, such as myopia. Each measurement is represented to highlight its role in understanding the structural dimensions of the eye.^[[2]](#footnote-2)^


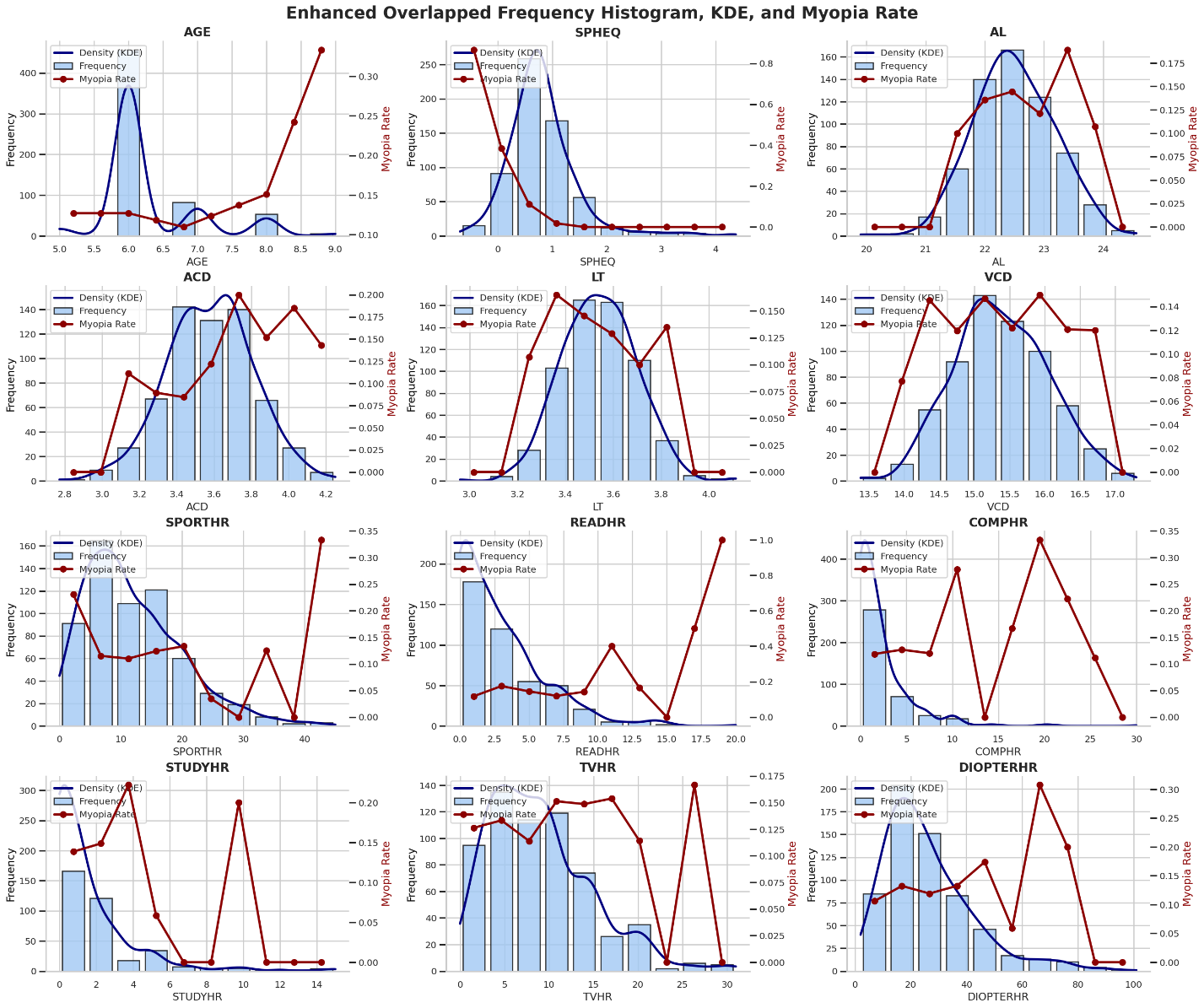


SupFig 2. Baseline characteristics of numerical variables of dataset1 (OLSM)


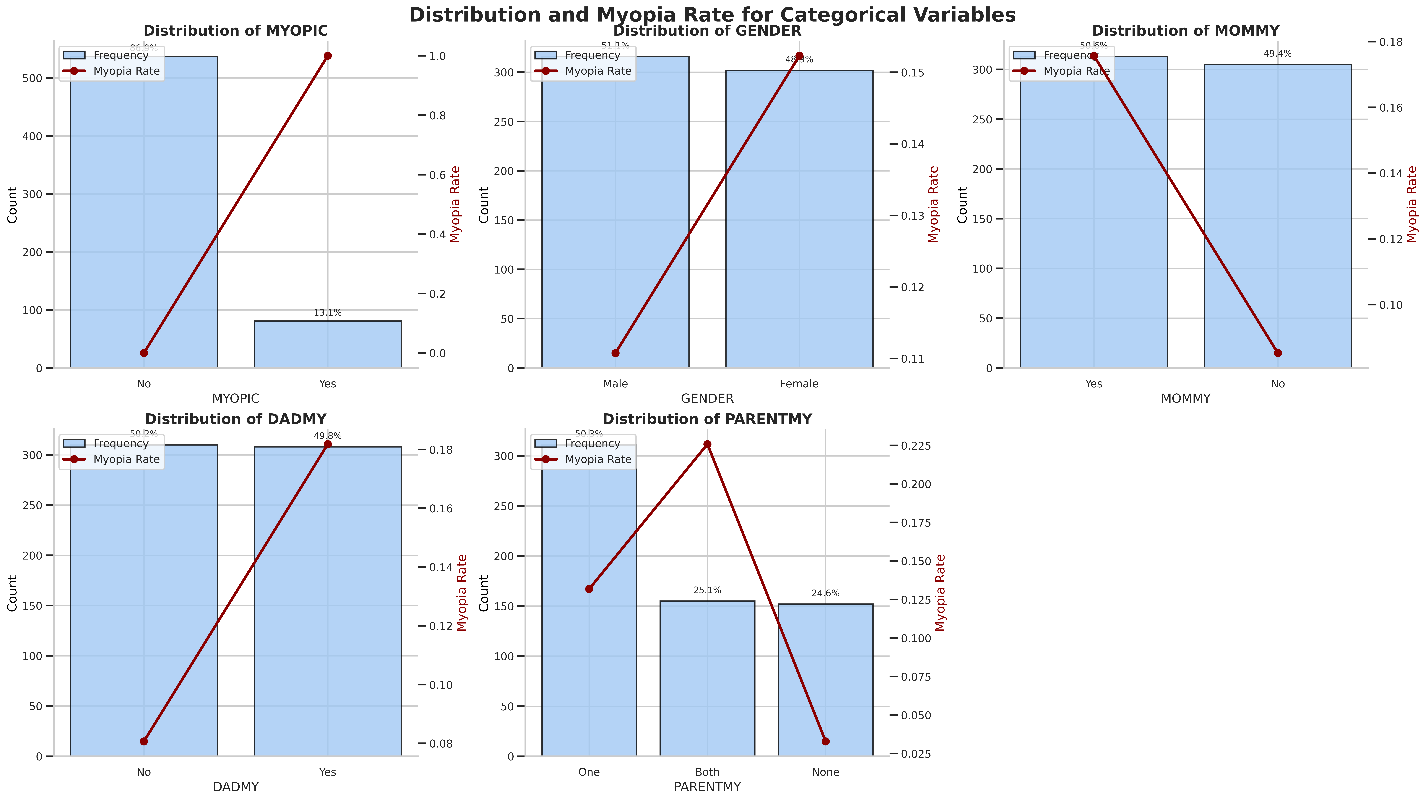


SupFig3. Distribution of categorical variables in dataset1 (OLSM)


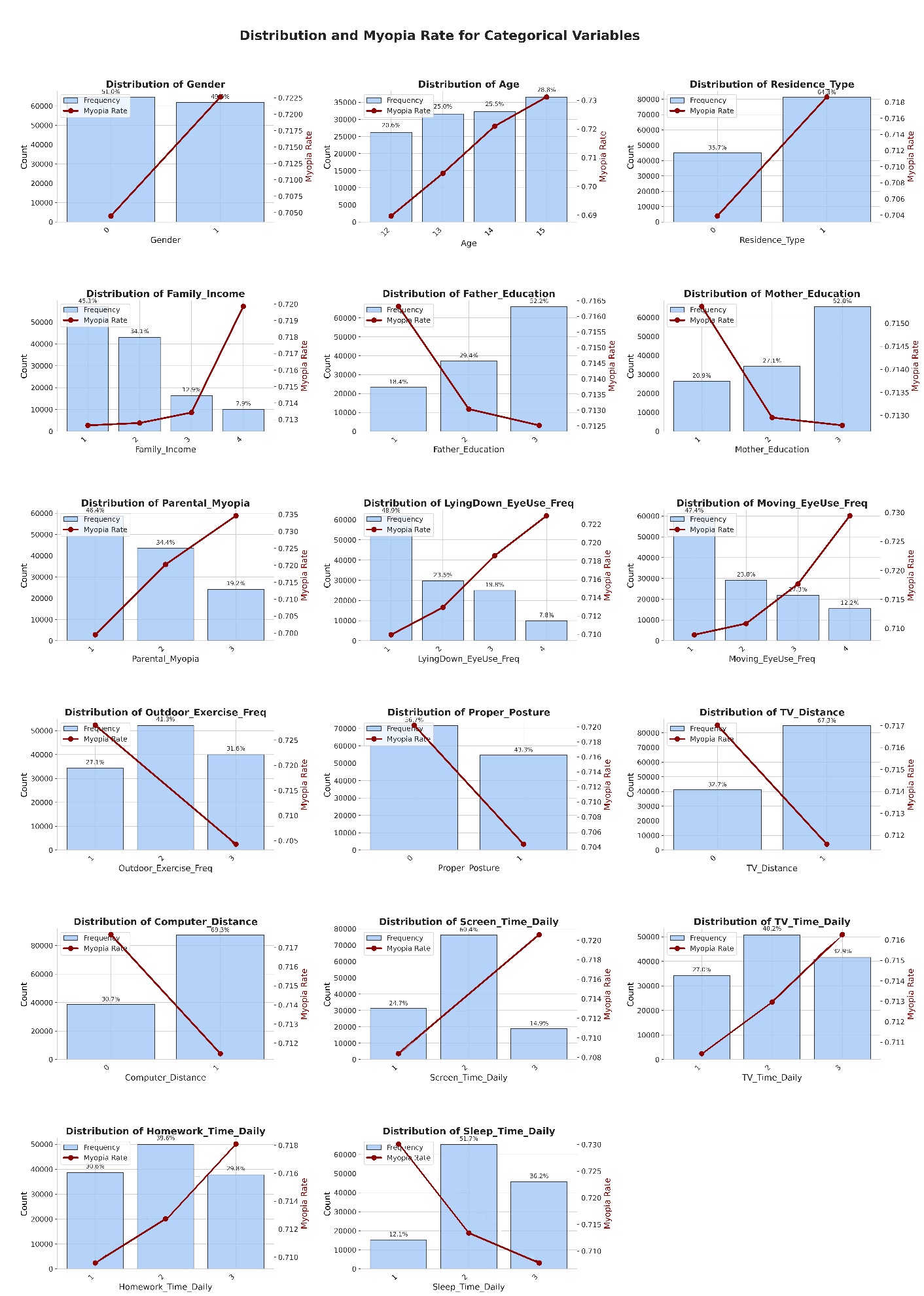


SupFig 4. The baseline characteristics of dataset 2 parameters and variables

1. https://github.com/ggeop/Myopia-Study [↑](#footnote-ref-1)
2. Figure from (https://visionscienceacademy.org/the-significance-of-measuring-axial-length-in-myopia/) [↑](#footnote-ref-2)
